# Ectopic insulinoma: case report

**DOI:** 10.1186/s12893-019-0661-y

**Published:** 2019-12-18

**Authors:** Mengqing Sun, Yaping Luo, Yan You, Xianlin Han, Yupei Zhao, Xianlin Han, Yupei Zhao

**Affiliations:** 1 Department of General Surgery, Peking Union Medical College Hospital, Beijing, China; 2 Department of Nuclear Medicine, Peking Union Medical College Hospital, Beijing, China; 3 Department of Pathology, Peking Union Medical College Hospital, Beijing, China

**Keywords:** Ectopic insulinoma, ^68^Ga-Exendin-4 PET/CT, Hypoglycemia

## Abstract

**Background:**

Ectopic insulinoma is a rare entity that is difficult to diagnose before surgery. This article reports two cases of ectopic insulinoma.

**Case presentation:**

Two patients manifested recurrent hypoglycemia with a typical Whipple triad. In terms of the qualitative diagnosis, the oral glucose tolerance test (OGTT) suggested a diagnosis of hyperinsulinemic hypoglycemia. However, preoperative imaging did not show a significant mass in the pancreas. In one patient, preoperative abdominal enhanced volume perfusion computed tomography (CT), somatostatin receptor imaging and ^99m^Tc-HYNIC-TOC SPECT/CT revealed a mass with a rich blood supply anterior to the duodenum. In the other patient, preoperative enhanced CT, magnetic resonance imaging (MRI) and ^68^Ga-Exendin-4 PET/CT showed a mass above the spleen. After surgical removal of the tumor, both patients received a confirmed diagnosis of neuroendocrine tumors by postoperative pathology. The symptoms of hypoglycemia were relieved after surgery, and the blood glucose level was significantly increased.

**Conclusion:**

Ectopic insulinoma is difficult to locate before surgery. ^68^Ga-Exendin-4 PET/CT has a high diagnostic value. Surgical removal of the lesion is main treatment.

## Background

Insulinoma is the most common functional pancreatic neuroendocrine tumor. It is usually a single, benign tumor with a low incidence [1]. Surgical removal of the lesion is the main treatment. More than 90% of patients can be cured by surgery [1]. The most important purpose of preoperative diagnosis is to localize the tumor. Ectopic insulinoma is derived from ectopic islet β-cells and is an extremely rare type of insulinoma. Because a conventional examination may not find a mass in the pancreas, this disease is difficult to diagnose and treat. In this study, we report two patients with ectopic insulinoma who were admitted to Peking Union Medical College Hospital. Both were surgically treated and achieved relief of recurrent hypoglycemia episodes.

## Case presentation

### Case 1

A 51-year-old woman was admitted to Peking Union Medical College Hospital in 2014 due to episodic loss of consciousness for 3 years and aggravation for 1 month. The patient started to have episodic loss of consciousness without a known cause during sleep (between 1 and 2 AM) 3 years prior. During the episodes, she was nonresponsive and had facial convulsions, sweating and hand shaking. Occasionally, she had episodes before lunch or dinner, accompanied by slow responses, dizziness and blurred vision. The frequency of the episodes was once every 2–3 weeks. Her blood glucose and electrolyte levels were not measured at the time of the attacks. There was no obvious abnormality in an enhanced magnetic resonance imaging (MRI) examination of the brain. The attack frequency had increased in the past month. Her fasting blood glucose levels were less than 2.8 mmol/L in multiple examinations. The symptoms subsided after eating. Her clinical manifestations were consistent with Whipple’s triad. A 3-h oral glucose tolerance test (3 h-OGTT) showed a blood glucose level of 1.6 mmol/L and an insulin level of 16.15 mU/L. Abdominal plain and enhanced computed tomography (CT) scans of the pancreas were unremarkable. The patient had gained 3 to 4 kg in the past 3 years. After admission, a 5 h-OGTT showed a blood glucose level of < 3 mmol/L, an insulin level of > 3 mU/L and a C-peptide level of > 0.6 ng/mL, with supporting hypoglycemia caused by excessive endogenous insulin (Table 1). In terms of multiple endocrine neoplasia type 1 (MEN-1) screening, anterior pituitary function, including levels of sex hormones, prolactin, growth hormone, thyroid-stimulating hormone (TSH), thyroid function, adrenocorticotropic hormone (ACTH) and cortisol, was not obviously abnormal. The patient’s parathyroid hormone (PTH) level was not elevated. Thus, there was no evidence for a diagnosis of MEN-1.

**Table 1:** 5 h-OGTT in Case 1

	Blood glucose (mmol/L)	Insulin (μIU/mL)	C-peptide (ng/mL)
0 h	2.5	30.74	2.98
0.5 h	4.5	134.51	5.72
1 h	5.2	117.83	7.92
2 h	5.2	32.06	4.62
3 h	3.3	14.44	2.39
4 h	2.4	16.03	2.02
5 h	2.0	22.03	2.50

**Fig. 1:**
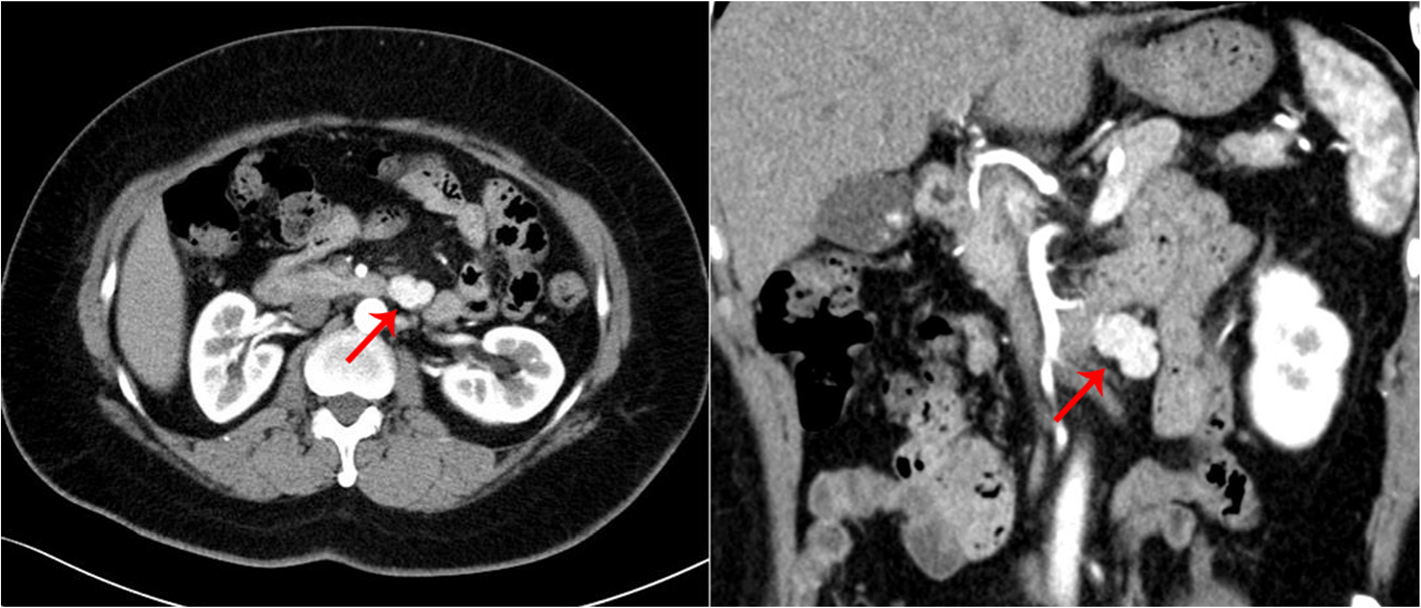
Case 1: VPCT revealed a lobulated mass with a rich blood supply anterior to the left anterior abdominal aorta, below the pancreatic body and anterior-inferior to the duodenum. This finding indicates a suspected diagnosis of ectopic insulinoma

**Fig. 2:**
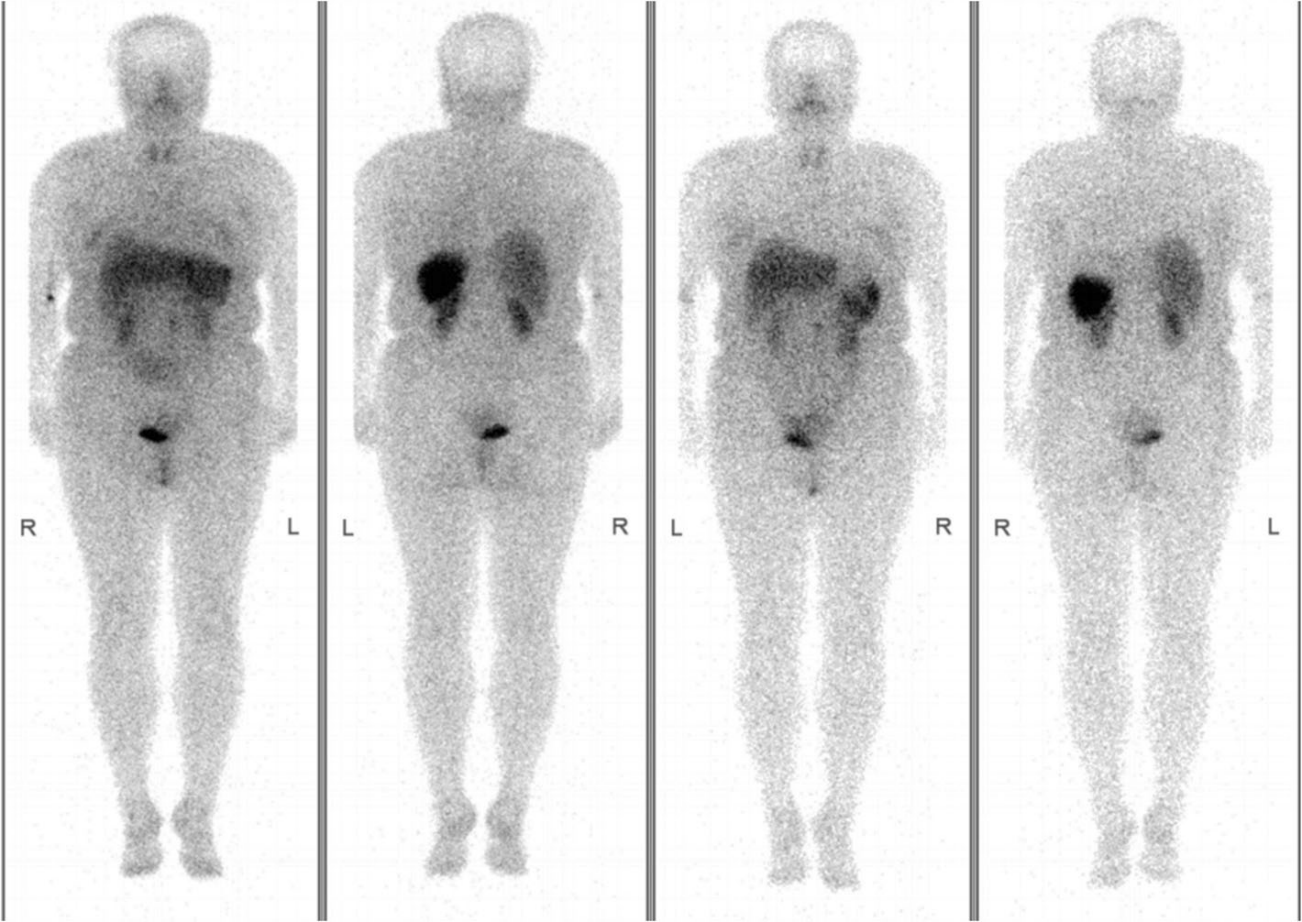
Case 1: somatostatin receptor imaging with 99mTc-HYNIC-TOC shows high expression of somatostatin receptors in the lesion located in the midline upper abdomen

**Fig. 3:**
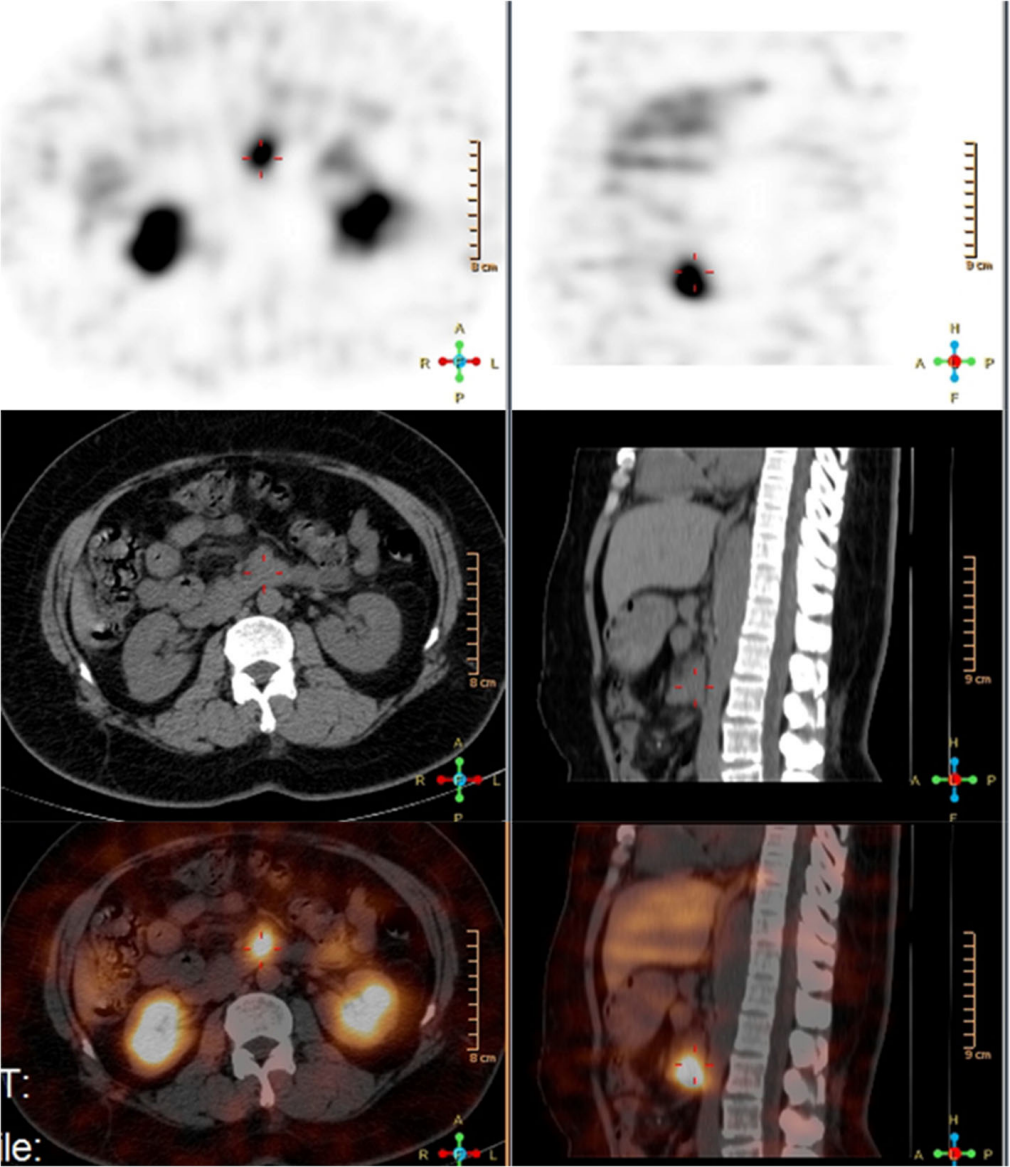
Case 1: 99mTc-HYNIC-TOC SPECT/CT shows increased radioactivity in the lesion anterior-inferior to the duodenum

Localization diagnosis: Pancreatic volume perfusion CT (VPCT) (Fig. 1) revealed a lobulated mass with a rich blood supply anterior to the left anterior abdominal aorta, below the pancreatic body and anterior-inferior to the duodenum, which indicated suspected ectopic insulinoma. The blood supply to the mass mainly originated from the superior mesenteric artery. Somatostatin receptor imaging with ^99m^Tc-HYNIC-TOC (Fig. 2) showed high expression of somatostatin receptors in the lesion located in the midline upper abdomen anterior-inferior to the duodenum, indicating neuroendocrine tumors without exclusion of lymph node metastasis. No significant abnormalities in somatostatin receptor expression were observed in the remaining tissues. ^99m^Tc-HYNIC-TOC SPECT/CT (Fig. 3) shows increased radioactivity in the lesion anterior-inferior to the duodenum.

**Fig. 4:**
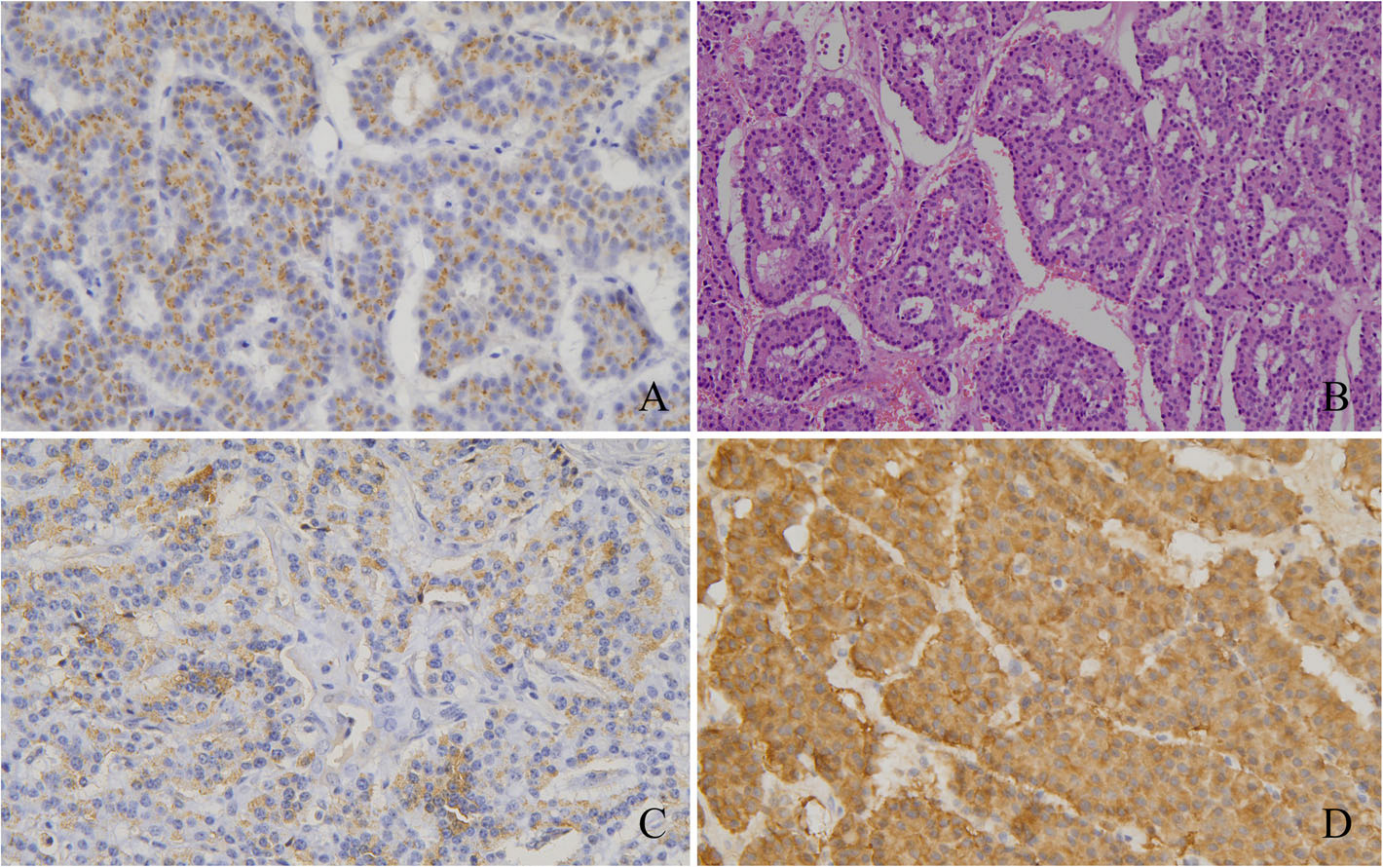
Case 1: postoperative pathology confirmed a diagnosis of neuroendocrine tumor. **a** CgA (+); **b** HE staining; **c** Insulin foci (+); **d** Syn (+)

After completion of the preoperative qualitative and localization diagnoses, the patient underwent robot-assisted ectopic islet cell tumor resection under general anesthesia. Postoperative paraffin-embedded tissue pathology (Fig. 4) showed (retroperitoneal mass) a neuroendocrine tumor (G1, mitotic figures: 1/10 high-power fields (HPF)). The immunohistochemical results showed the following antibody expression: CD56 (NK-1) (weak +), CgA (+), gastrin (+), glucagon (−), insulin (spot +), Ki-67 (index approximately 2%), somatostatin (−), Syn (+), AE1/AE3 (+) and CD10 (−). The patient’s fasting blood glucose level increased to between 6.91 and 10.9 mmol/L after surgery.

### Case 2

A 37-year-old woman was admitted to Peking Union Medical College Hospital in 2018 due to intermittent confusion for 2 years. Two years prior, the patient had a sudden episode of confusion with gazing of both eyes and inability to correctly answer question before eating dinner. Her blood glucose level was not measured at this time. After 30 min, she recovered spontaneously from unconsciousness. Since then, her symptoms had included apathy, slow response and inability to correctly answer repeated questions (once every 2 months) after working for a long time or before dinner. Her blood glucose level was not measured. One year prior, the patient repeatedly had sudden loss of consciousness after activities accompanied by sweating. Her blood glucose level was lower than the lowest limit of the measurable value, and she gradually regained consciousness after receiving an intravenous injection of a “high concentration of glucose”. Her symptoms were consistent with the Whipple triad. A further examination was performed, which showed the following: blood glucose level 1.37 mmol/L, insulin level 6.69 μIU/mL and C-peptide level 1.99 ng/mL. Enhanced MRI and abdominal ultrasound did not detect any mass in the pancreas. Then, the patient started eating regular snacks between meals and monitoring her blood glucose level, which was usually between 2 and 4 mmol/L. The number of hypoglycemia episodes before meals was significantly reduced. A 3 h-OGTT was performed after admission, and the results are shown in Table 2. In terms of MEN-1 screening, her anterior pituitary function, including sex hormones, prolactin, growth hormone, TSH, thyroid function, ACTH and cortisol, was not obviously abnormal. Her PTH level was not elevated. Thus, no evidence existed for a diagnosis of MEN-1.

**Table 2:** 3 h-OGTT in Case 2

	Blood glucose (mmol/L)	Insulin (μIU/mL)	C-peptide (ng/mL)
0 h	3.85	2.16	0.7
1 h	9.01	25.13	5.45
2 h	5.75	9.98	5.45
3 h	2.48	4.94	2.08

**Fig. 5:**
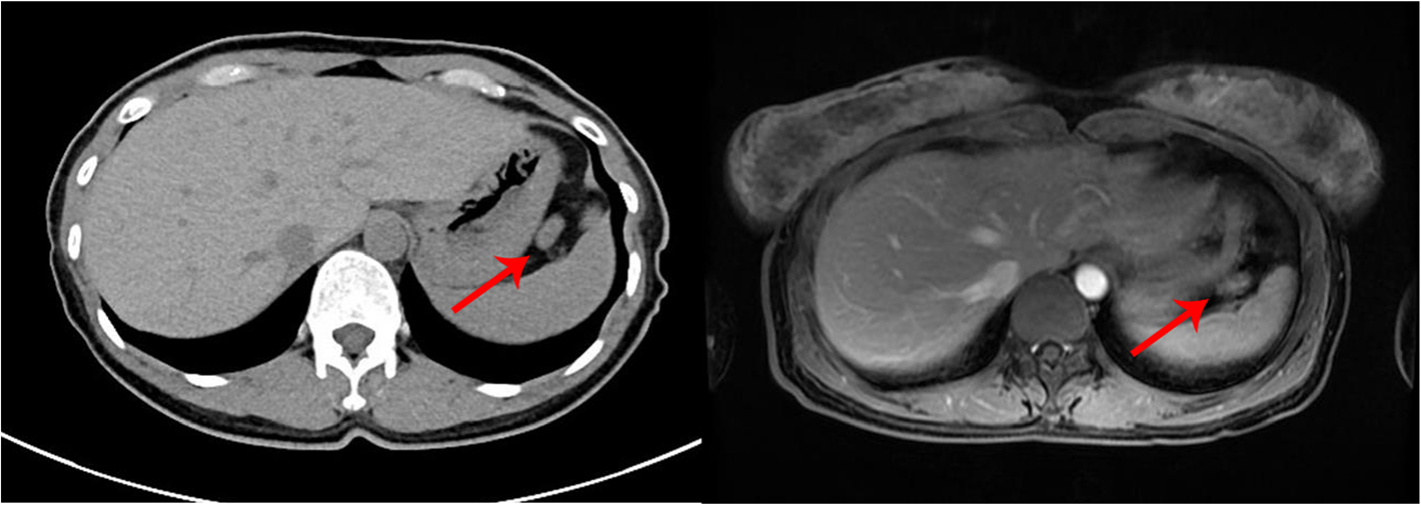
Case 2: enhanced CT (left) and high-resolution MRI (right) of the pancreas showed no obvious mass or high-perfusion nodules within the pancreas, but a nodule was observed above the spleen, which was considered an accessory spleen

**Fig. 6:**
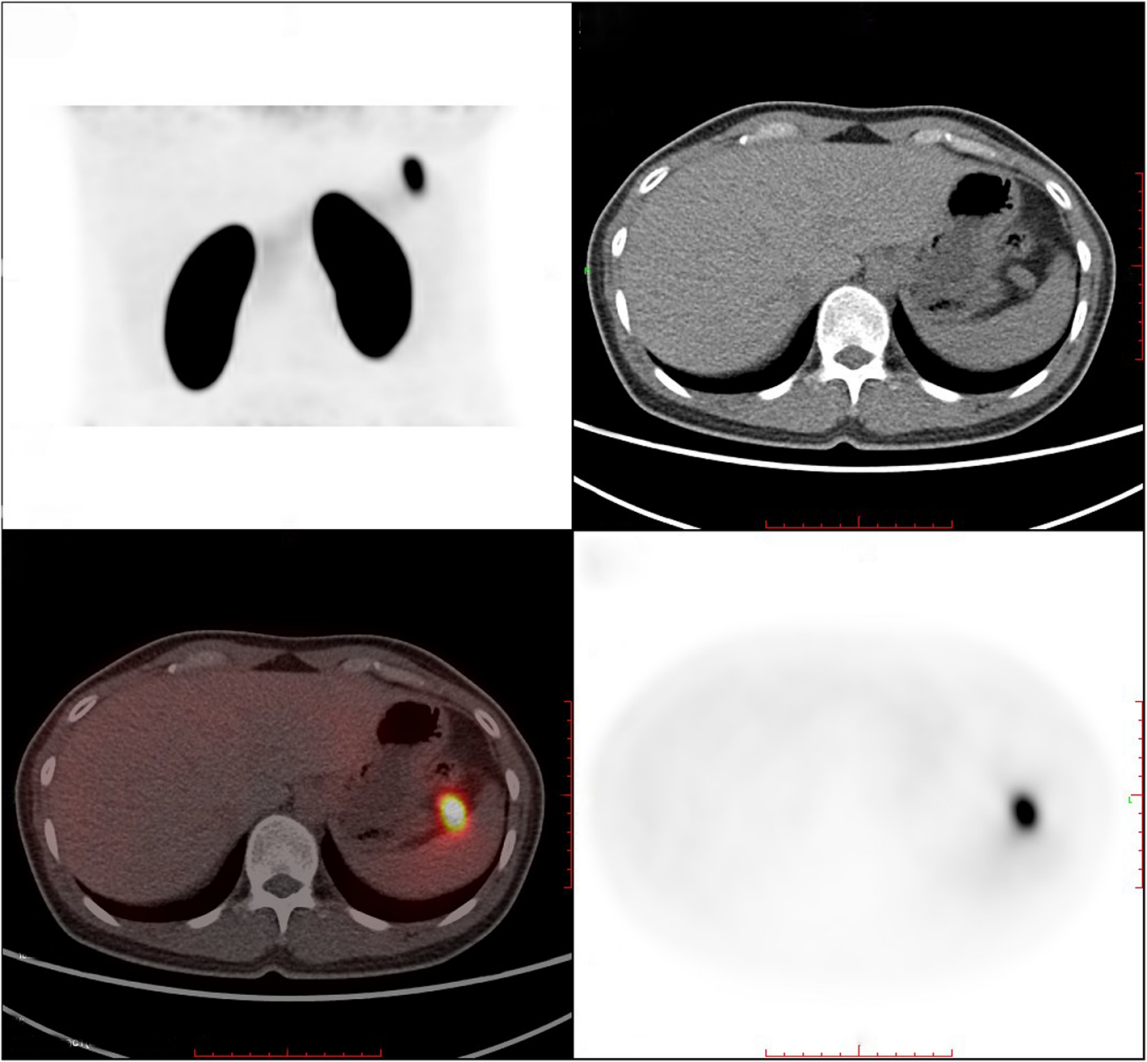
Case 2: 68Ga-Exendin-4 PET/CT showing intense uptake in the nodule near the splenic hilum, suggesting an insulinoma

**Fig. 7:**
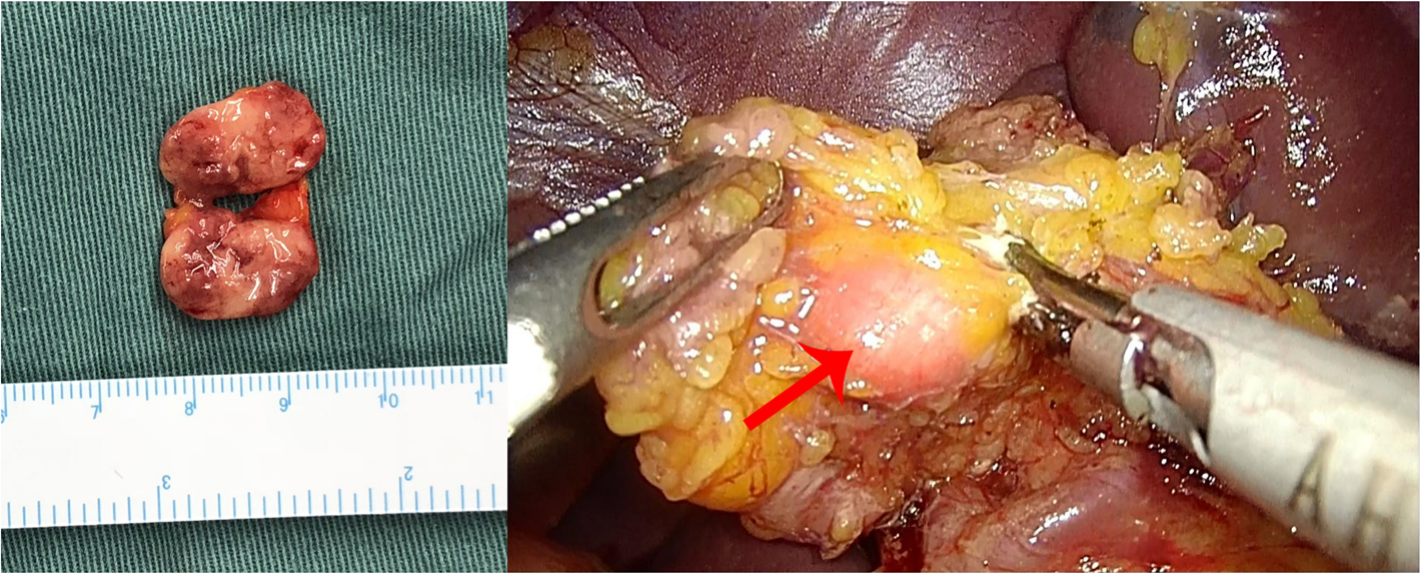
Case 2: Left: gross examination of surgical specimen. Right: intraoperative findings showing a tumor located in the gastrosplenic ligament and close to the spleen. Local resection of the tumor was performed

**Fig. 8:**
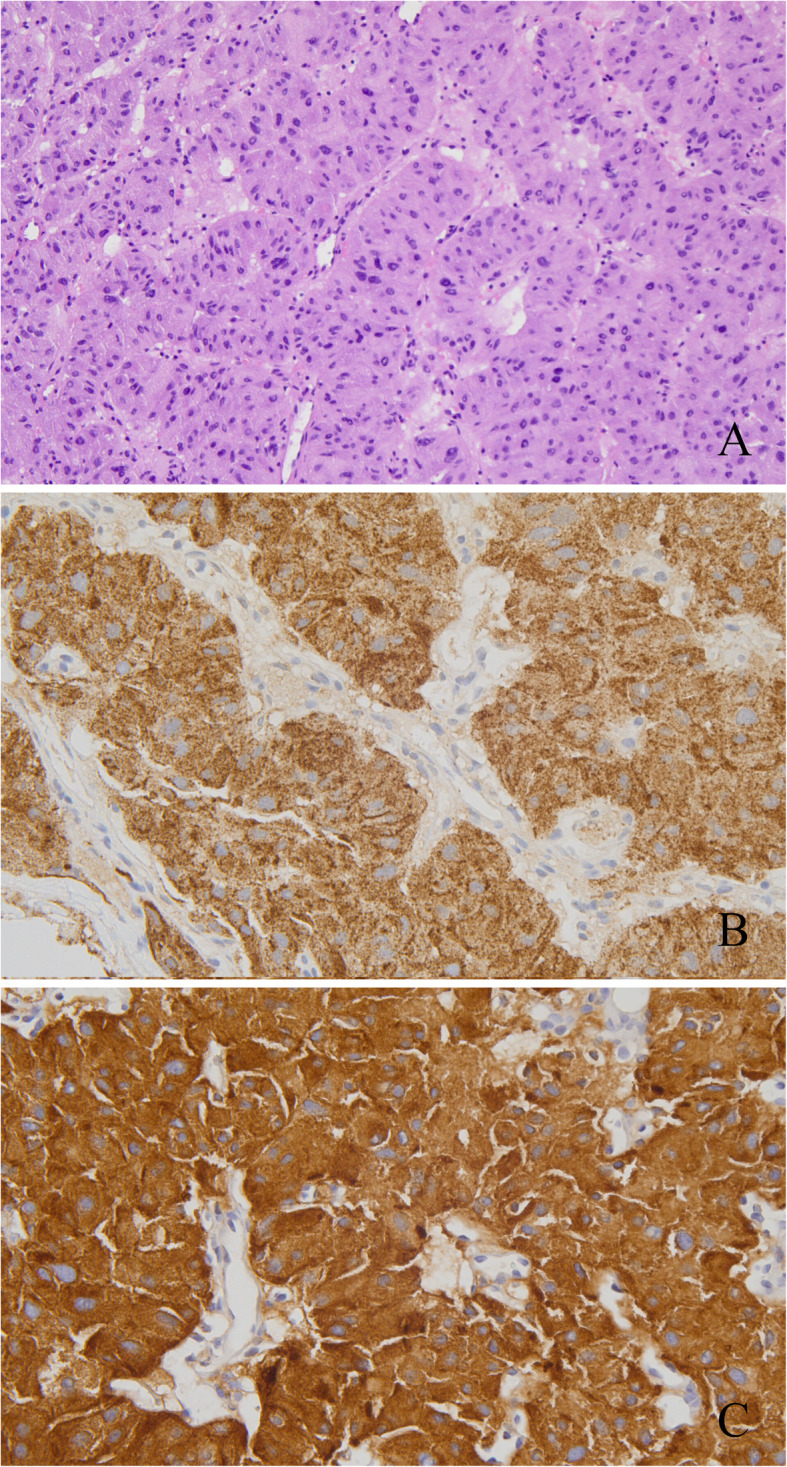
Case 2: Postoperative pathology confirmed a diagnosis of neuroendocrine tumor. **a** HE staining; **b** CgA (+); **c** Syn (+)

In terms of the localization diagnosis, somatostatin receptor imaging did not show high somatostatin receptor expression levels, i.e., no signs of a neuroendocrine tumor. Enhanced CT, VPCT and high-resolution MRI of the pancreas (Fig. 5) showed no obvious mass or high-perfusion nodules within pancreas, but a nodule was observed above the spleen, indicating the possibility of an accessory spleen. ^68^Ga-Exendin-4 PET/CT (Fig. 6) showed abnormal radioactive uptake in the tail of the pancreas (near the spleen). These findings, combined with her medical history, indicated a diagnosis of insulinoma. Considering comprehensive studies including ^68^Ga-Exendin-4 PET/CT, pancreatic enhanced CT, VPCT and enhanced MRI, we diagnosed the patient with an ectopic insulinoma located in the omental tissue of the gastrosplenic ligament above the spleen. Laparoscopy was performed to explore lesions, release adhesion and remove the ectopic insulinoma (Fig. 7). After surgery, the patient’s blood glucose level returned to the normal range. Postoperative tissue pathology (Fig. 8) showed neuroendocrine tumors (G2, mitotic figures: 1/10 HPF). The immunohistochemistry results showed the following antigen expression: melan-A (−), AE1/AE3 (+), CgA (+), Ki-67 (index 3%), S-100 (−), α-inhibin (−), Syn (+) and CK7 (−). Her fasting blood glucose level increased to 5.9 mmol/L after surgery.

## Discussion and conclusions

Insulinoma is the most common functional pancreatic neuroendocrine tumor derived from islet β-cells. The literature summarizes its characteristics according to 90% statements: 90% of insulinomas are single adenomas, 90% are benign, 90% are < 2 cm in diameter, and 90% are located in the pancreas [2]. Ectopic insulinoma is a rare entity, with only a few cases reported in the literature. The diagnosis of this disease is difficult, and even repeated surgical exploration could show negative findings [3]. We searched English publications in PubMed from the last 20 years and found eight patients with this disease. Their data are summarized in Table 3.

**Table 3:** Summary of patients with ectopic insulinoma reported in the literature and in this study (2 patients)

	Year	Authors	Sex/age	Grade	Tumor location	Metastasis	Preoperative localization	Size	Procedure
	2004	Tolentino et al. [4]	M/23	N/M	duodenum	N/A	CT and EGDS (esophagogastroduodenoscopy)	2.5 cm	locally excised
2	2005	Hennings et al. [5]	F/74	N/M	adjacent to the duodenum (Treitz ligament)	N/A	somatostatin receptor scintigraphy including a single photon emission computed tomography (SPECT) study	3 cm	enucleation
3	2009	Cardenas et al. [6]	M/46	N/M	intrasplenic heterotopic pancreas	LN metastasis	MRI	7.6 × 4.4 cm	splenectomy
4	2011	Xian-Ling et al. [7]	M/21	N/M	duodenohepatic ligament	N/A	CT and DSA (digital subtraction angiography)	1 cm	wedge resection of the tumor
5	2013	La Rosa et al. [8]	F/75	G1	duodenum	N/A	CT and EGDS	1.5 cm	enucleation
6	2015	Liu et al. [9]	F/79	N/M	retroperitoneum under the hepatoduodenal ligament	N/A	CT	2.5 cm	enucleation
7	2018	Li et al. [10]	F/53	G1	pelvis	five lesions in the pelvis	^68^Ga-DOTA-NOC-PET/CT	N/M	removal of the five lesions
8	2018	Liu et al. [11]	F/31	G1	proximal jejunum	multiple liver metastasis	CT and ^68^Ga-Exendin-4 PET/CT	3.5 cm	everolimus plus long- acting SSA octreotide
9	2019	Case 1	F/51	G1	adjacent to the duodenum	N/A	CT, somatostatin receptor scintigraphy and ^99m^Tc-HYNIC-TOC SPECT/CT	2.5 cm	locally excised
10	2019	Case 2	F/37	G2	gastrosplenic ligament	N/A	CT, MRI, ^68^Ga -Exendin-4-PET/CT	1.2 cm	locally excised

Regarding the definition of ectopic insulinoma, no precise definition has been provided in the literature. The case reports roughly include several categories. The first category is insulinoma comprising an ectopic pancreas. This category is the least controversial. An ectopic pancreas is a rare congenital anomaly. The incidence of this condition on autopsy in the literature is approximately 0.5–13.7%. The common sites of an ectopic pancreas include the stomach, duodenum, colon and Meckel’s diverticulum [12]. The ectopic insulinomas reported in this study were located near the pancreas, most commonly in the duodenum. This condition is also reported in the duodenal ligament, spleen, Treitz ligament and proximal jejunum. The tumor location in this study was generally consistent with reports in the literature. Second, nonfunctional gastrointestinal neuroendocrine tumors may also secrete insulin during the course of the disease, causing symptoms similar to insulinoma. Li et al. [10] reported a case with five ectopic tumors that were located in the pelvic cavity. This situation is very rare. This patient had a history of rectal nonfunctional neuroendocrine tumor resection. They suggested that the ectopic tumors may be secondary to the rectal nonfunctional neuroendocrine tumor [10]. It is uncertain whether this condition was defined as ectopic insulinoma. In addition, Clover et al. [13] reported a case of nonfunctional malignant neuroendocrine tumor with insulin secretion after sunitinib treatment. However, the case report caused controversy. Third, other extrapancreatic tumors have been reported to secrete insulin and cause hyperinsulinemic hypoglycemia, including ovarian tumors, cervical carcinoids, kidney carcinoids, paragangliomas and liver neuroendocrine tumors [14, 15]. In this study, these tumors were not classified as an ectopic insulinoma. In addition, other extrapancreatic tumors may secrete insulin-like growth factors (IGF-1/IGF-II), such as leiomyosarcoma and liver tumors, though they do not originate from islet cells or secrete insulin. Thus, these tumors cannot be called insulinomas [5, 16].

The Whipple triad is the classic clinical manifestation of insulinoma. However, in addition to hypoglycemia and the Whipple triad, ectopic insulinoma can cause other symptoms related to its location. For instance, insulinoma in the duodenum can cause gastrointestinal bleeding, jaundice and abdominal pain [4, 17]. Most insulinomas are less than 2 cm in diameter, but more than half of the ectopic insulinomas we have reviewed from the literature are greater than 2 cm (6/10) in diameter. This difference may be related to delayed diagnosis and the long course of the disease. Moreover, one patient had a large heterogeneous mass, i.e., the mass included considerable adipose tissue in addition to islet cells [6]. The two patients reported here had tumor sizes of 2.5 cm and 1.2 cm, which is consistent with the size of orthotopic insulinomas.

Approximately 3–5% of patients with insulinoma may also have MEN-1. Patients reported by Liu et al. [11] in 2018 were suspected of having MEN-1 because of a mild elevation of PTH that returned to a normal level during follow-up. A diagnosis of MEN-1 was not confirmed in the patients in this study or in other patients reported in the literature. Therefore, ectopic insulinoma has not been found to be associated with MEN-1.

Interestingly, the term “malignant insulinoma” [5, 6, 10, 11] was used in four cases reported in the literature (4/8). This term is different than our understanding that most “insulinomas” are benign. However, the definition of malignant insulinoma in these studies is very vague. One patient showed a neuroinvasive lesion microscopically. One patient had a multifocal lesion. One patient presented with splenic infiltration and lymph node metastasis. Only one patient had definite liver metastasis. It is generally believed that malignant insulinoma can be differentiated from benign insulinoma mainly by local invasion and distant metastasis [18]. Therefore, only two of the above cases can be confirmed as malignant insulinoma. Neither of the patients we reported to have malignant manifestations. However, the overall incidence of ectopic insulinoma is too low; therefore, conclusions regarding the malignant tendency of ectopic insulinoma should be carefully considered**.**

The difficulty of preoperative diagnosis of insulinoma lies in the localization diagnosis, which is the key to the success of surgical treatment. However, imaging studies may not show any mass in the pancreas in patients with ectopic insulinoma. The low incidence of this disease and the insufficient awareness of many doctors are associated with the difficulty of the preoperative localization diagnosis. In general, the sensitivity of the noninvasive examinations commonly used in the diagnosis is approximately 56–70% for CT and 63–86% for MRI [18]. However, in patients with ectopic insulinoma, even if preoperative CT and MRI can identify lesions, it is still difficult to determine whether the lesion is insulinoma because it is not located in the pancreas. The imaging findings cannot determine the relationship between the lesion and hypoglycemia. Lesions found solely on CT or MRI can only be identified as insulinomas that cause hypoglycemia by postoperative pathology and the glycemic response [6, 9]. Nuclear medicine examinations including somatostatin receptor imaging and ^68^Ga-NOTA-Exendin-4 PET/CT have greater advantages in determining the types and function of tumors. However, the sensitivity rate of somatostatin receptor imaging is very low; the rate reported in the literature is only 19.5–50% [18, 19]. ^68^Ga-NOTA-Exendin-4 PET/CT is currently the most sensitive noninvasive test, with a sensitivity rate of 97.7% [19], and is of great value for the diagnosis of ectopic insulinoma. Of the eight patients reported in the literature we reviewed, three were diagnosed by somatostatin receptor imaging or ^68^Ga-NOTA-Exendin-4 PET/CT. In this study, Case 1 was diagnosed with ectopic insulin because the positive findings determined by somatostatin receptor imaging were consistent with those indicated by VPCT. These observations help further confirm the diagnosis of ectopic insulinoma. In Case 2, preoperative enhanced CT and MRI showed the ectopic insulinoma mass but suggested a diagnosis of an “accessory spleen” because the mass was located close to the spleen. Enhancement with contrast made the differential diagnosis difficult. Positive imaging with ^68^Ga-Exendin-4 PET/CT was the key to the complete localization diagnosis. In addition, the stomach and duodenum are common sites of ectopic insulinoma; therefore, some patients can be diagnosed by endoscopy combined with CT or endoscopic biopsy. It should be noted that due to the low incidence of this disease, even if the preoperative localization diagnosis of ectopic insulinoma is made, examination of the whole pancreas with intraoperative ultrasound or palpitation is still required to avoid a missed diagnosis of insulinoma. If the diagnosis and location are confirmed, surgical removal is the main treatment for ectopic insulinomas in most cases. The principle of treatment is the same as that of orthotic insulinomas. The key to surgical treatment is to completely remove all lesions. If no residual tumor is present after surgery, the patient’s hypoglycemia symptoms will be significantly relieved. In the two patients we reported, the postoperative blood glucose level increased significantly.

This study reports two cases of ectopic insulinoma in which patients exhibited recurrent hypoglycemia with a typical Whipple triad. Laboratory tests supported a diagnosis of hyperinsulinemic hypoglycemia. In addition to traditional noninvasive enhanced CT, PVCT and MRI, in recent years, preoperative localization diagnosis has also relied on somatostatin receptor imaging, ^99m^Tc-HYNIC-TOC SPECT/CT and ^68^Ga-Exendin-4 PET/CT to locate lesions outside the pancreas. Both patients were cured by surgical removal of the tumor. The incidence of this disease is low. According to a literature review, we found that common sites of insulinoma include the stomach, duodenum, colon and Meckel’s diverticulum. The Whipple triad is the major clinical manifestation. Gastrointestinal symptoms including hemorrhage, jaundice and abdominal pain may be present if the insulinoma involves the corresponding organs. Insulinoma has not been shown to have a specific association with MEN-1. Ectopic insulinomas appear to be larger than orthotic insulinoma and are associated with a slightly higher malignancy ratio. However, this finding lacks strong supportive data. The difficulty of preoperative diagnosis lies in localizing the tumor. ^68^Ga-Exendin-4 PET/CT has a high diagnostic value for this disease. Surgical removal of the lesion is main treatment.

## Data Availability

All patient data and clinical images adopted are contained in the medical files of Peking Union Medical College Hospital. The data supporting the conclusions of this article are included within the article and its figures and tables.

## Abbreviations

ACTH: Adrenocorticotropic hormone
CT: Computed tomography
DSA: Digital subtraction angiography
EGDS: Esophagogastroduodenoscopy
HPF: High-power fields
IGF: Insulin-like growth factors
MEN: Multiple endocrine neoplasia
MRI: Magnetic resonance imaging
OGTT: Oral glucose tolerance test
PET: Positron Emission Tomography
PTH: Parathyroid hormone
SPECT: Single-Photon Emission Computed Tomography
SRI: Somatostatin receptor imaging
TSH: Thyroid-stimulating hormone
VPCT: Volume perfusion computed tomography
